# Supplementary material for: A Comprehensive Analysis of Chloroplast Genome Provides New Insights into the Evolution of the Genus Chrysosplenium
Source: Int J Mol Sci. 2023 Sep 29;24(19):14735. doi: 10.3390/ijms241914735 (PMC10572340; doi:10.3390/ijms241914735)
Supplement: Supplementary file 1 [file ijms-24-14735-s001.zip › Supplementary Figure.pdf]

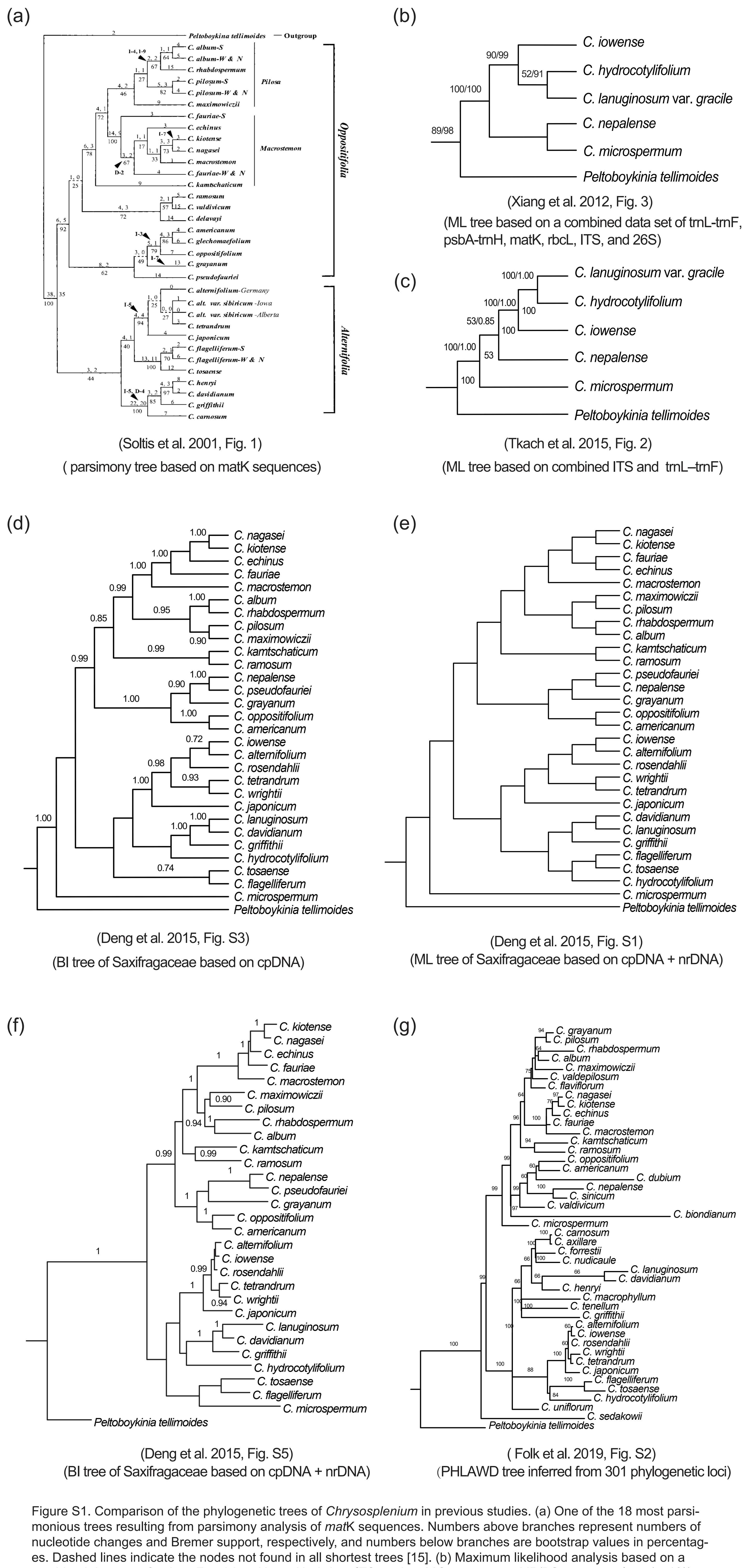

Figure S1. Comparison of the phylogenetic trees of *Chrysosplenium* in previous studies. (a) One of the 18 most parsimonious trees resulting from parsimony analysis of *mat*K sequences. Numbers above branches represent numbers of nucleotide changes and Bremer support, respectively, and numbers below branches are bootstrap values in percentages. Dashed lines indicate the nodes not found in all shortest trees [15]. (b) Maximum likelihood analysis based on a combined data set of *trn*L-*trn*F, *psb*A-*trn*H, *mat*K, *rbc*L, nrITS, and rrn26S sequences. MP/BS support values (>50%) are in-dicated above or below the branches [16]. (c) Maximum likelihood (ML) cladogram of *Micranthes* and representative members of other genera of Saxifragaceae based on combined nrITS and *trn*L-*trn*F sequence. ML bootstrap support and posterior probability values of ≥ 50% are shown above the branches, maximum parsimony (MP) bootstrap support of ≥ 50% below the branches [17]. (d) BI tree of Saxifragaceae based on cpDNA derived from MrBayes analysis. Numeric values at the nodes are Bayesian posterior probabilities, and PP values ≥ 0.70 are shown [18]. (e) ML tree of Saxifragaceae based on cpDNA+nrDNA based on RAxML analysis [18]. (f) BI phylogram tree of Saxifragaceae based on cpDNA+nrDNA with branch lengths. PP values ≥ 0.90 are shown [18]. (g) Complete PHLAWD tree, with original branch lengths measured in per-site substitution rate. Branch labels are bootstrap frequencies, which are only shown on branches with support ≥ 50% [19];

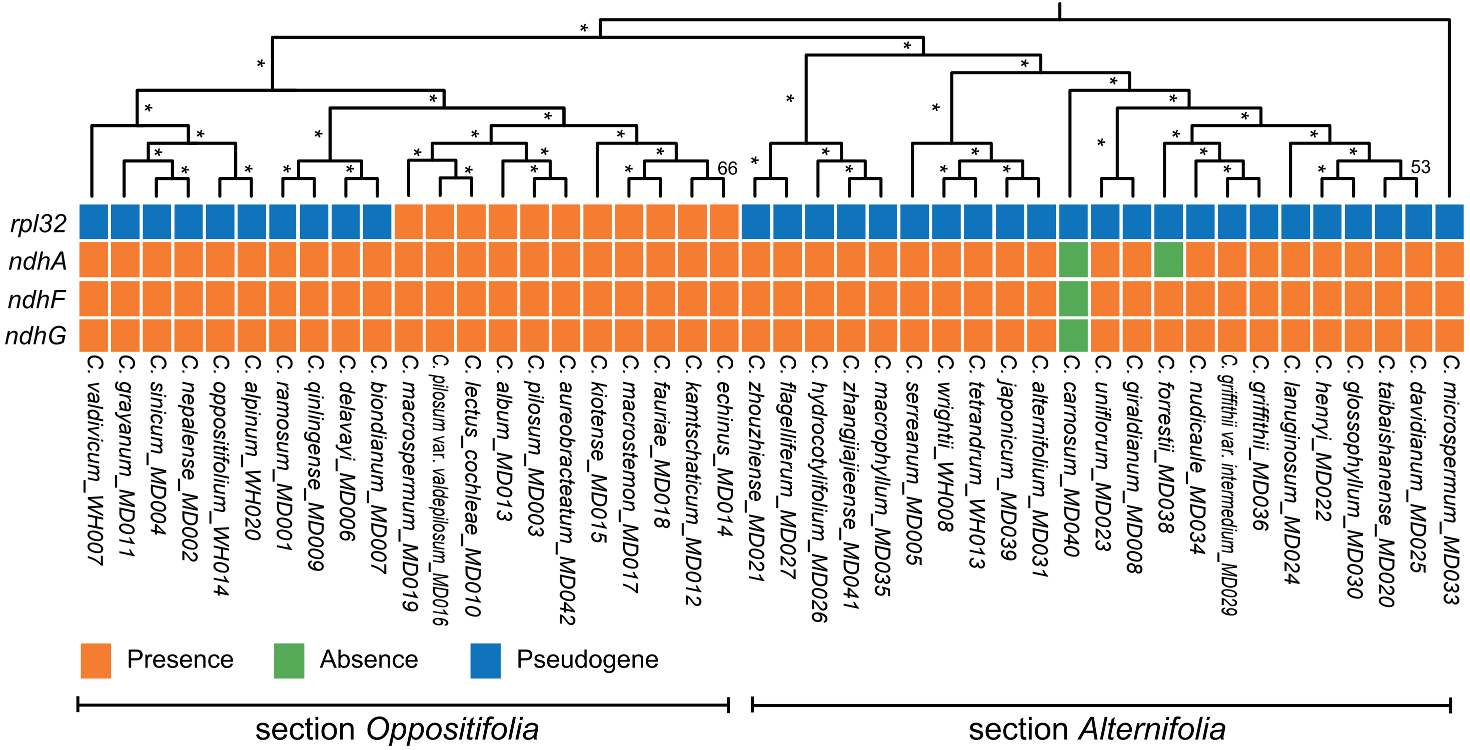

Figure S2. Statistics of four PCGs in the chloroplast genome of *Chrysosplenium* species. The values on the nodes indicate the ML bootstrap support values. The asterisks indicate the bootstrap support values of 100.

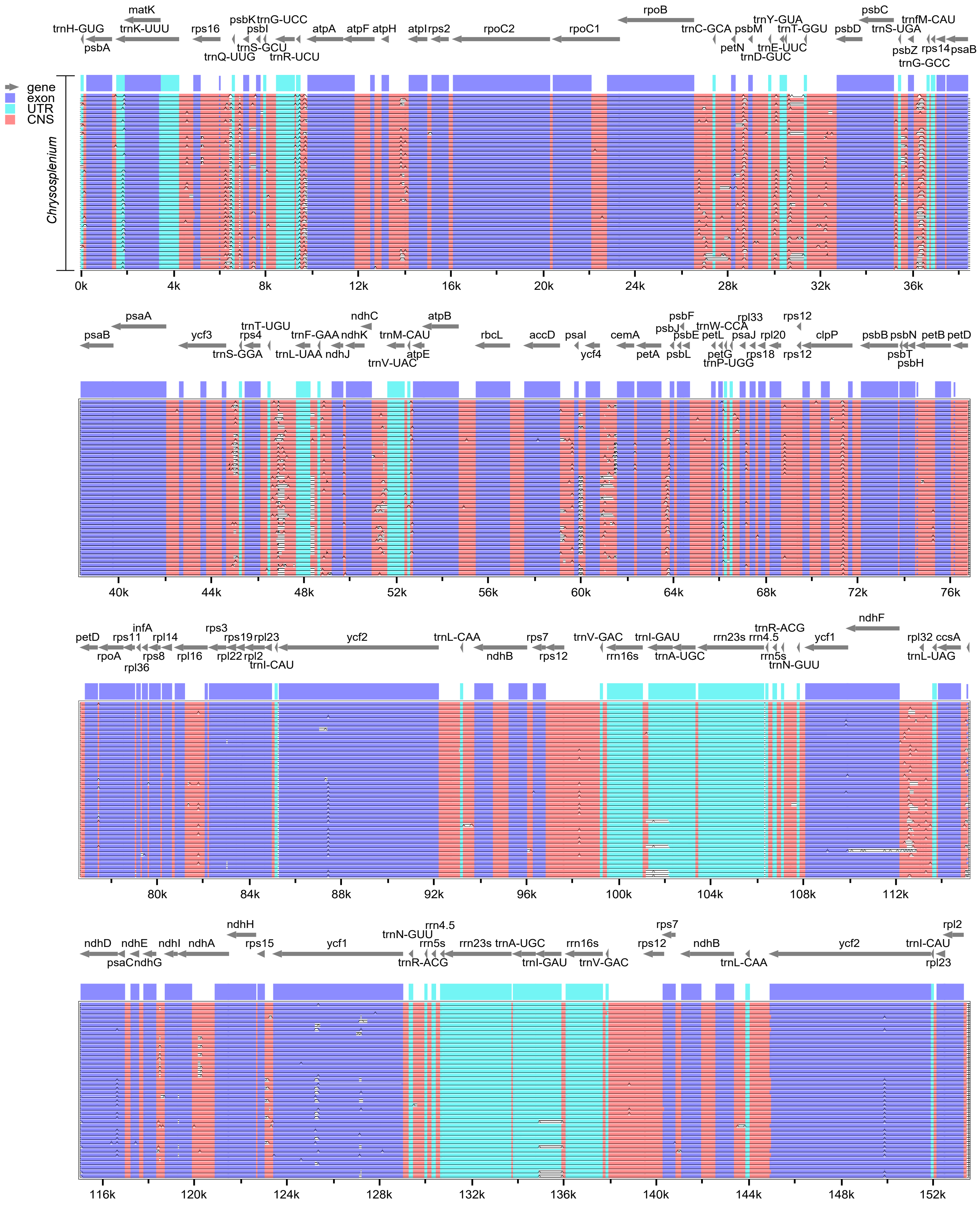

Figure S3. Plots of percent sequence identity of the cp genomes of 44 *Chrysosplenium* species with *C. ramosum* as a reference. The plots were visualized in mVISTA.

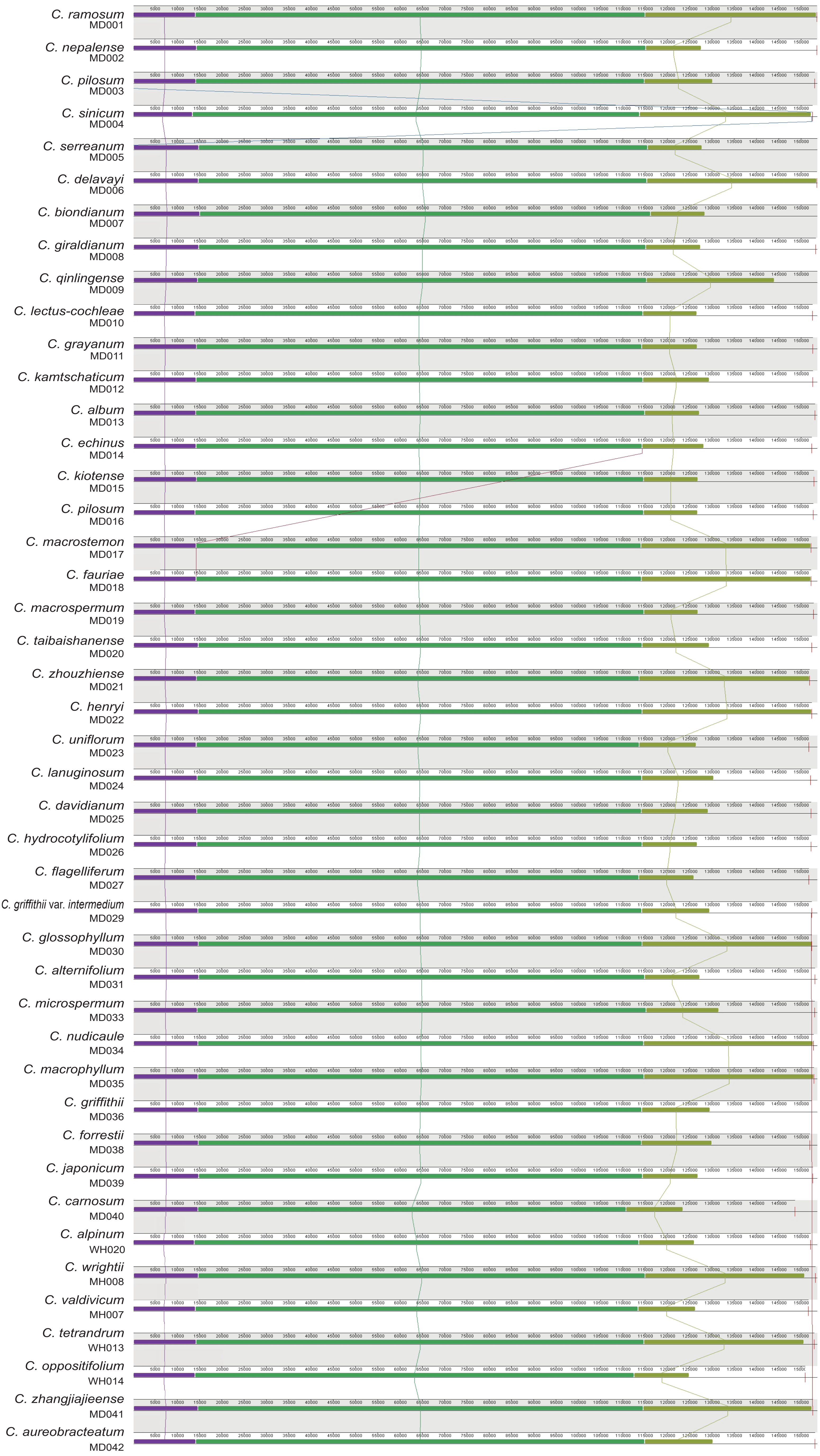

Figure S4. Mauve alignment of the chloroplast genomes of *Chrysosplenium* species. The long squares show genomic similarity, while the lines connecting them represent a covariate association.

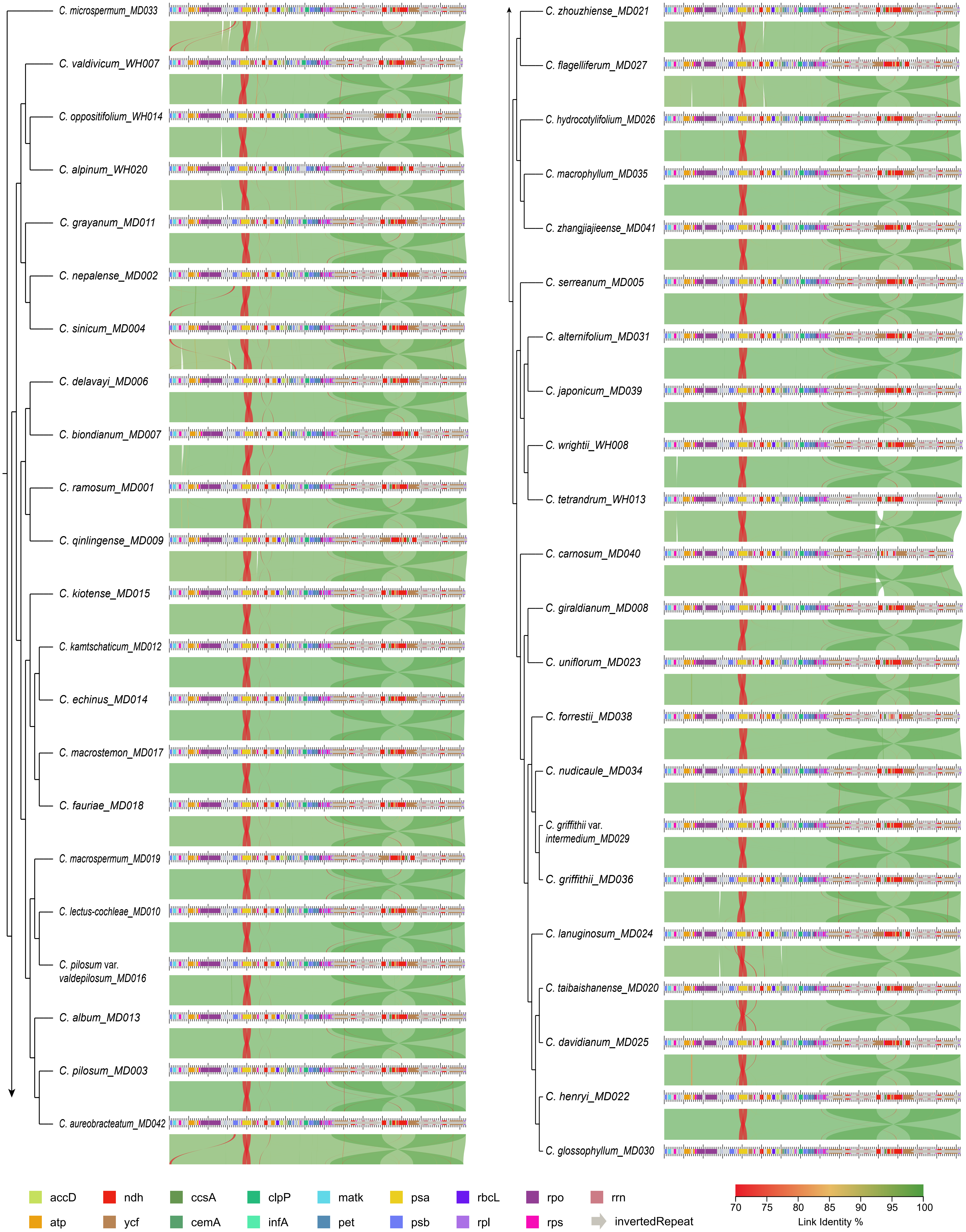

Figure S5. Genomic collinearity analysis of 44 *Chrysosplenium* species. Genes on the genome are represented by different colors. The gray blue color indicates the entire chloroplast genome region, the white arrowheads indicate the IR region of the chloroplast genome, and the line connecting them indicates the colinear relationship. Chaining relationships are indicated in red to green from low to high.

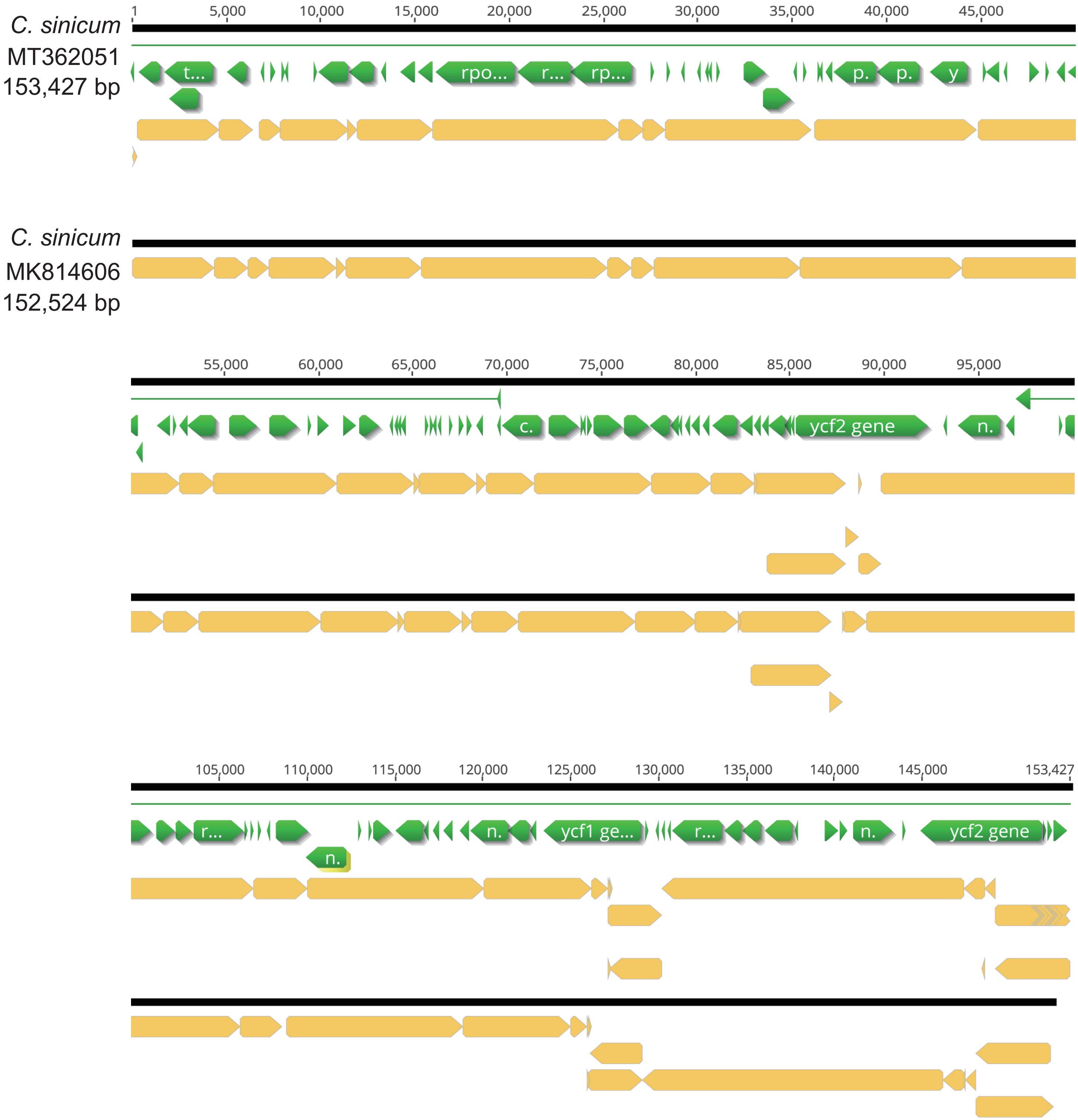

Figure S6. Comparison of chloroplast genomes within species of *C. sinicum*. Green boxes indicates genes, orange boxes indicates identical sequences.

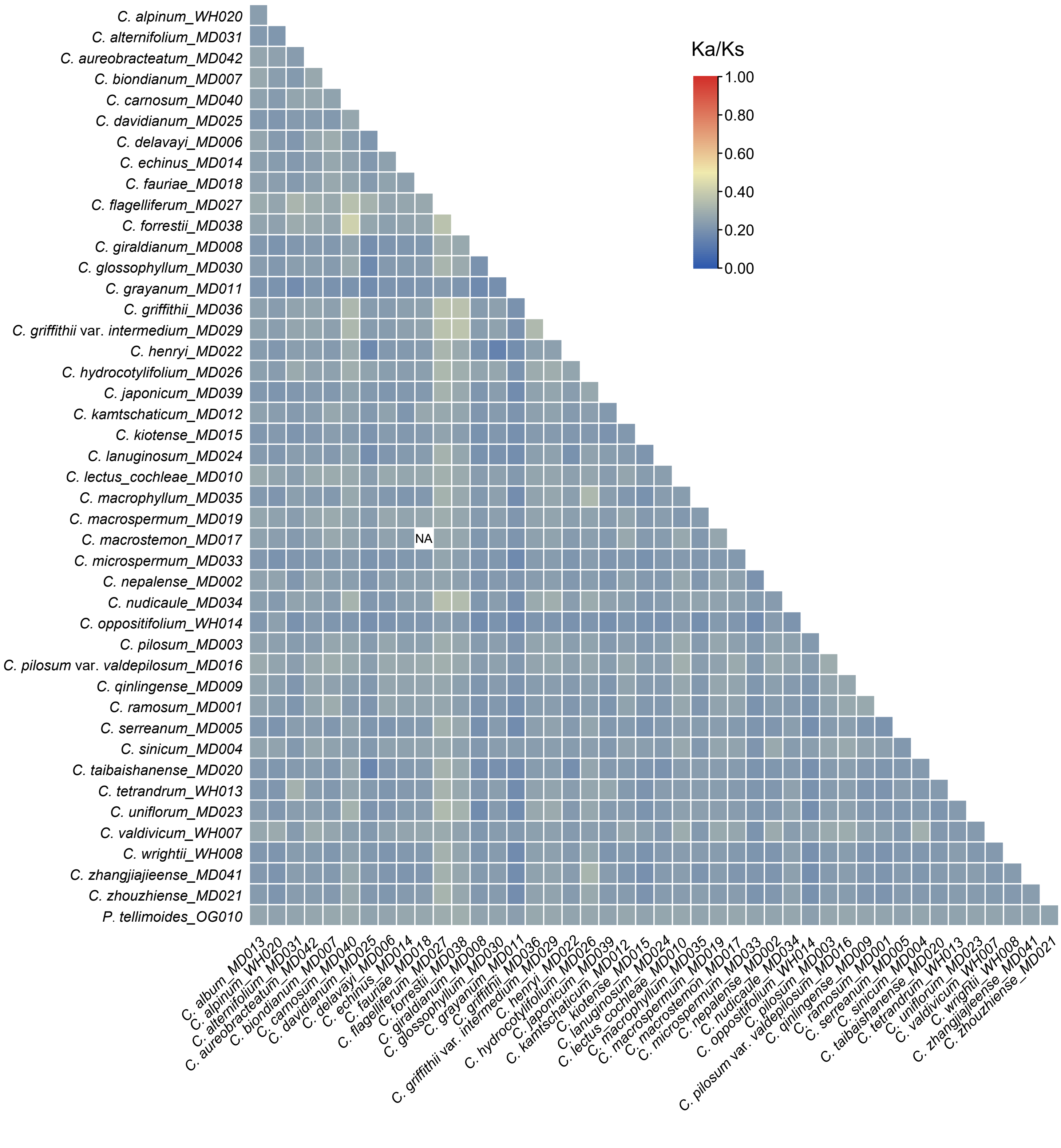

Figure S7. Selection pressure analysis based on species-level. The Ka/Ks ratios are shown in blue and red, from low to high, respectively.

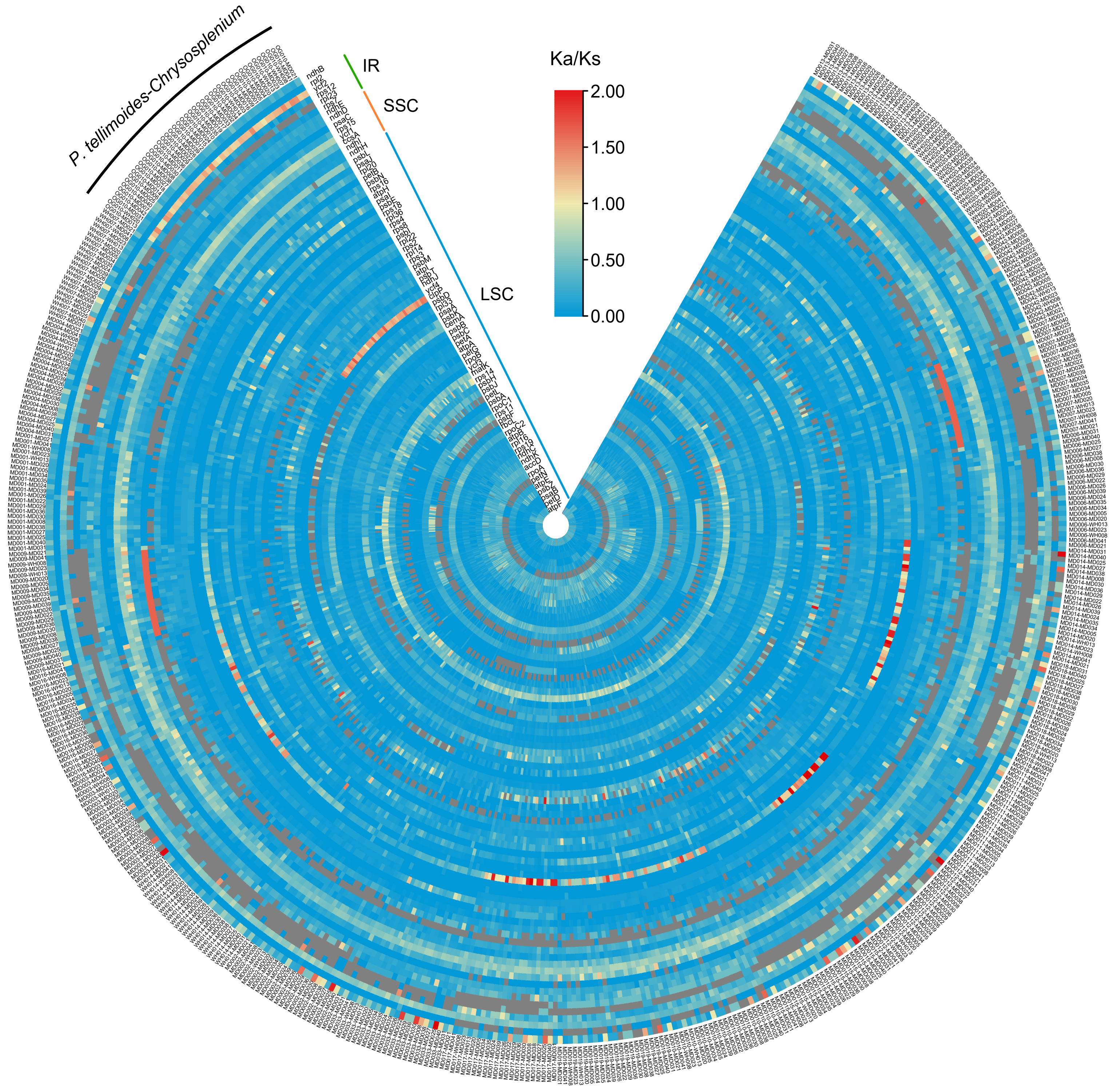

Figure S8. Selection pressure analysis between *Alternifolia* and *Oppositifolia* and between *Chrysosplenium* and *P. tellimoides*. The Ka/Ks ratios are shown in blue and red, from low to high, respectively.

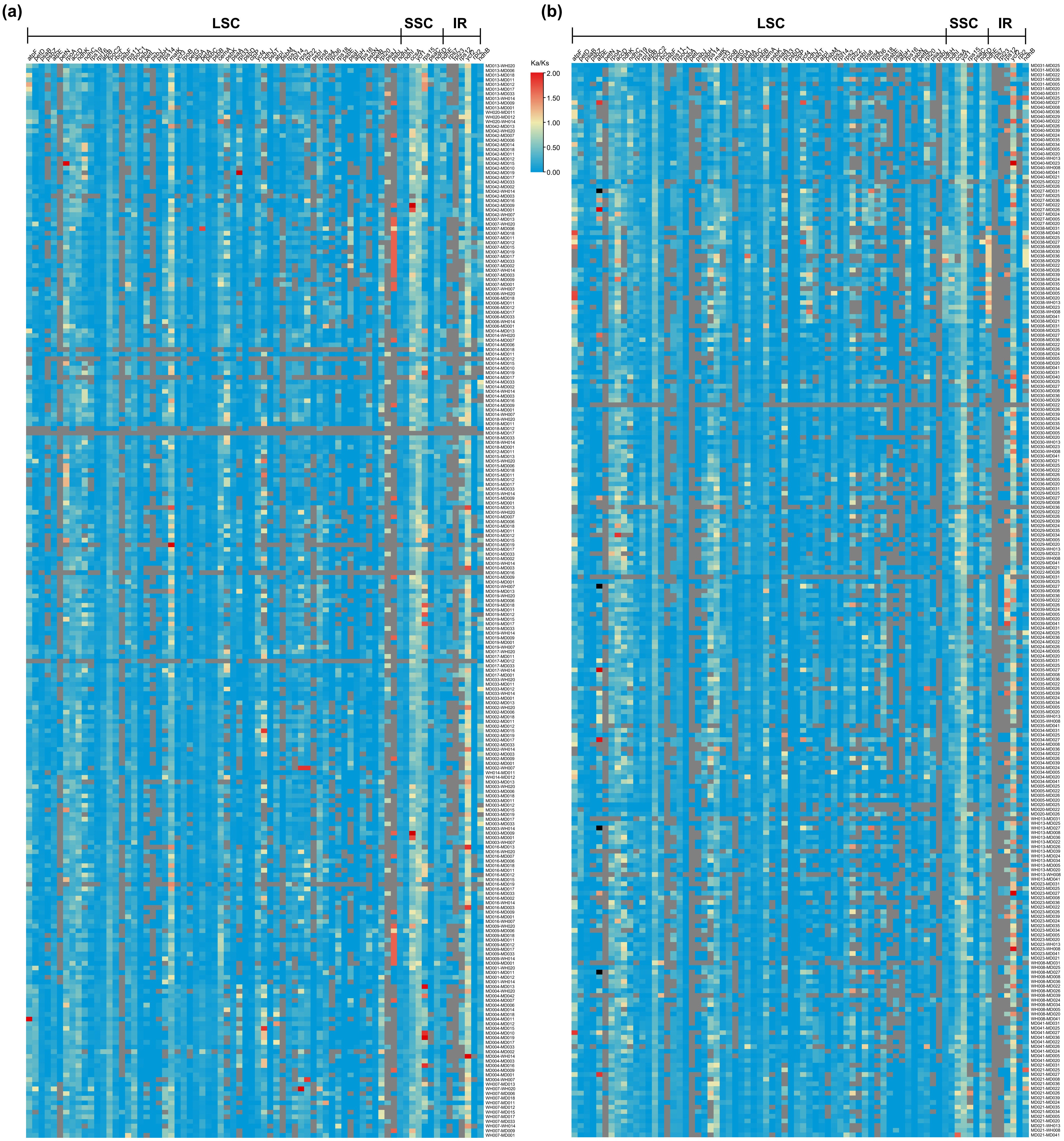

Figure S9. Selection pressure analysis of species within two sections in *Chrysosplenium*. (a) Selection pressure analysis on *Oppositifolia* species. (b) Selection pressure analysis on *Alternifolia* species; The Ka/Ks ratios are shown in blue and red, from low to high, respectively.

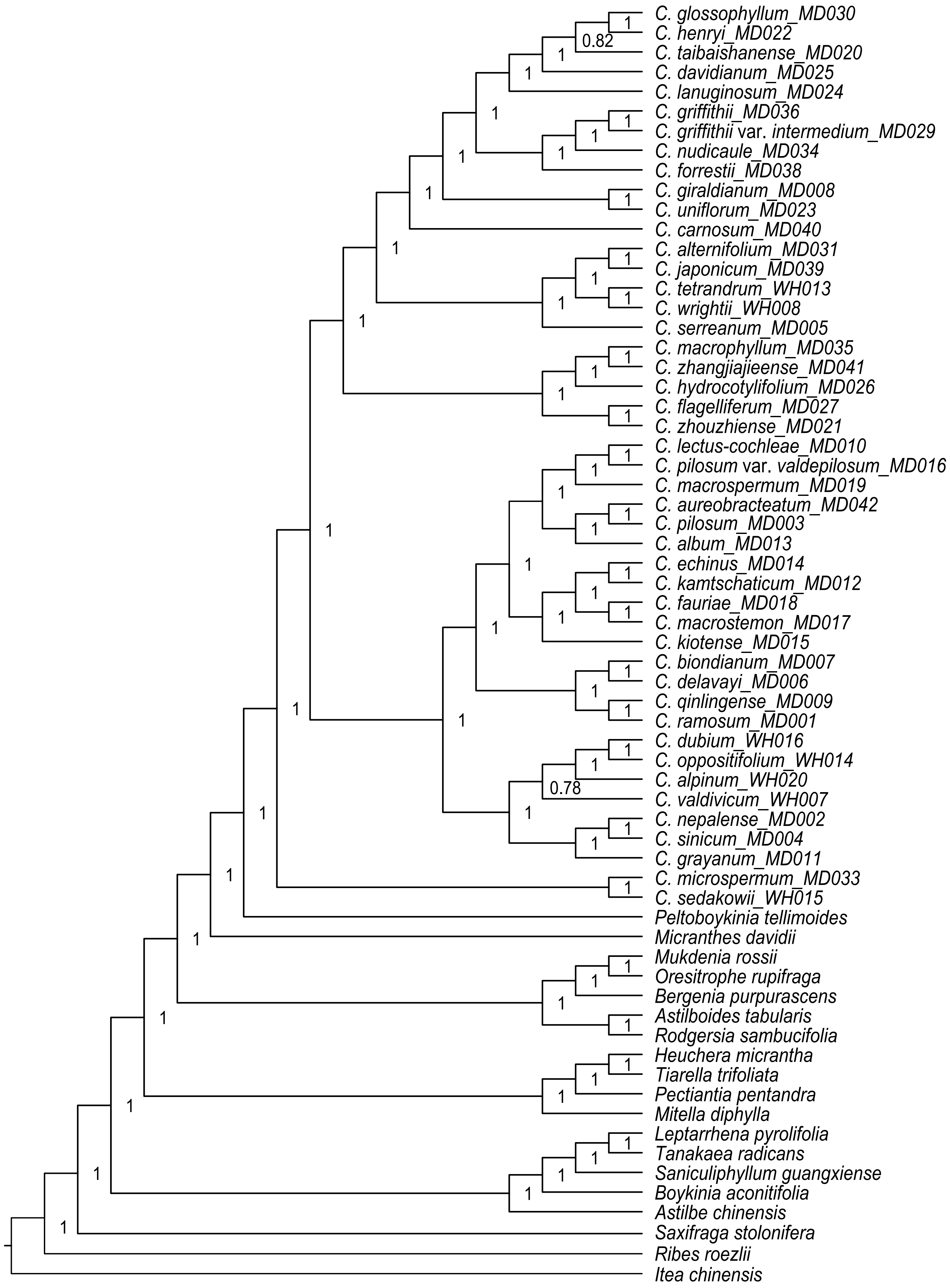

Figure S10. Phylogenetic tree of *Chrysosplenium* species using Bayesian inference (BI) analyses based on cpPCGs matrix; The values on the nodes indicate the BI posterior probabilities.

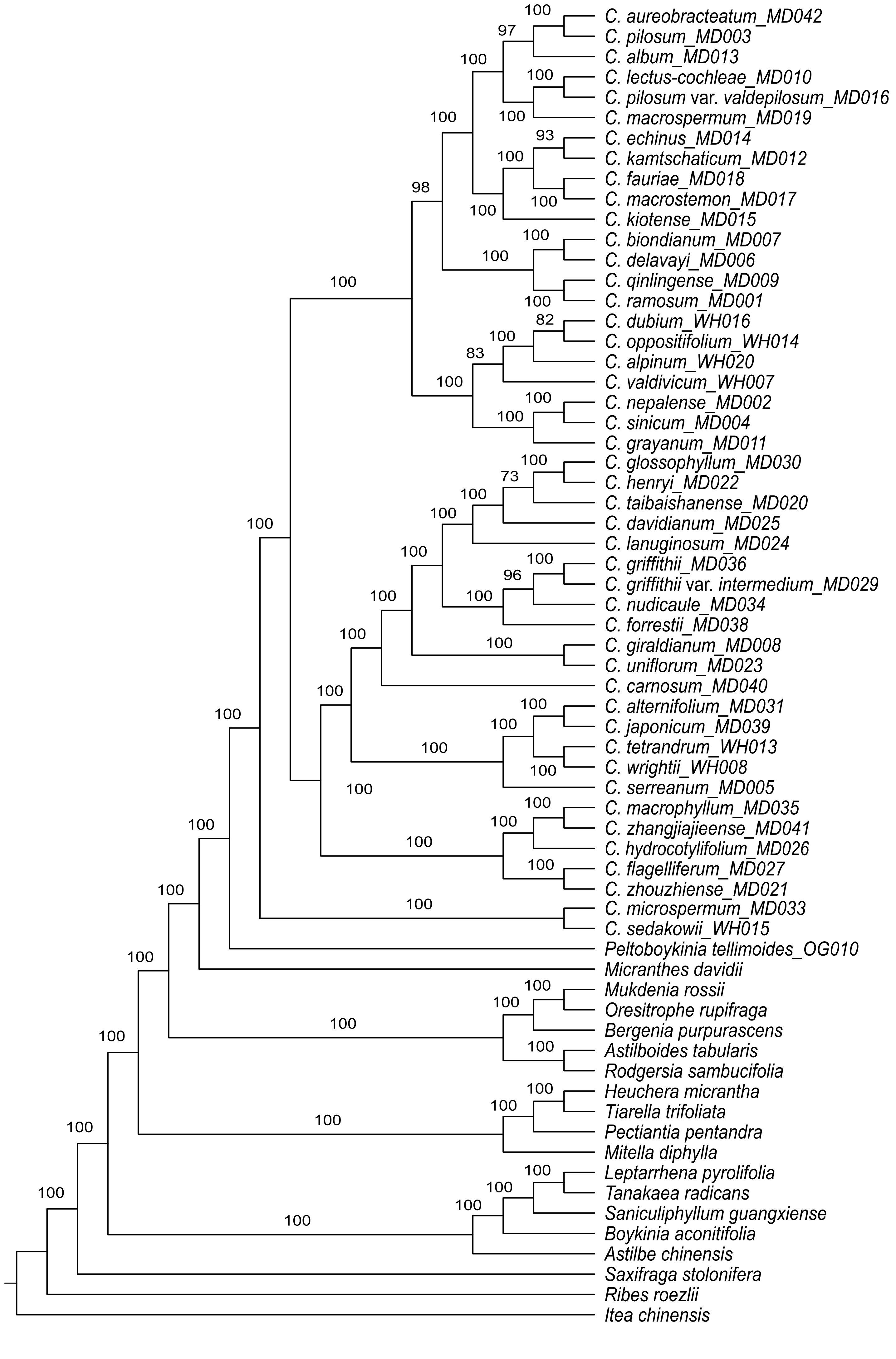

Figure S11. Phylogenetic tree of *Chrysosplenium* species using Maximum likelihood (ML) analyses based on cpPCGs matrix; The values on the nodes indicate the ML bootstrap support values.

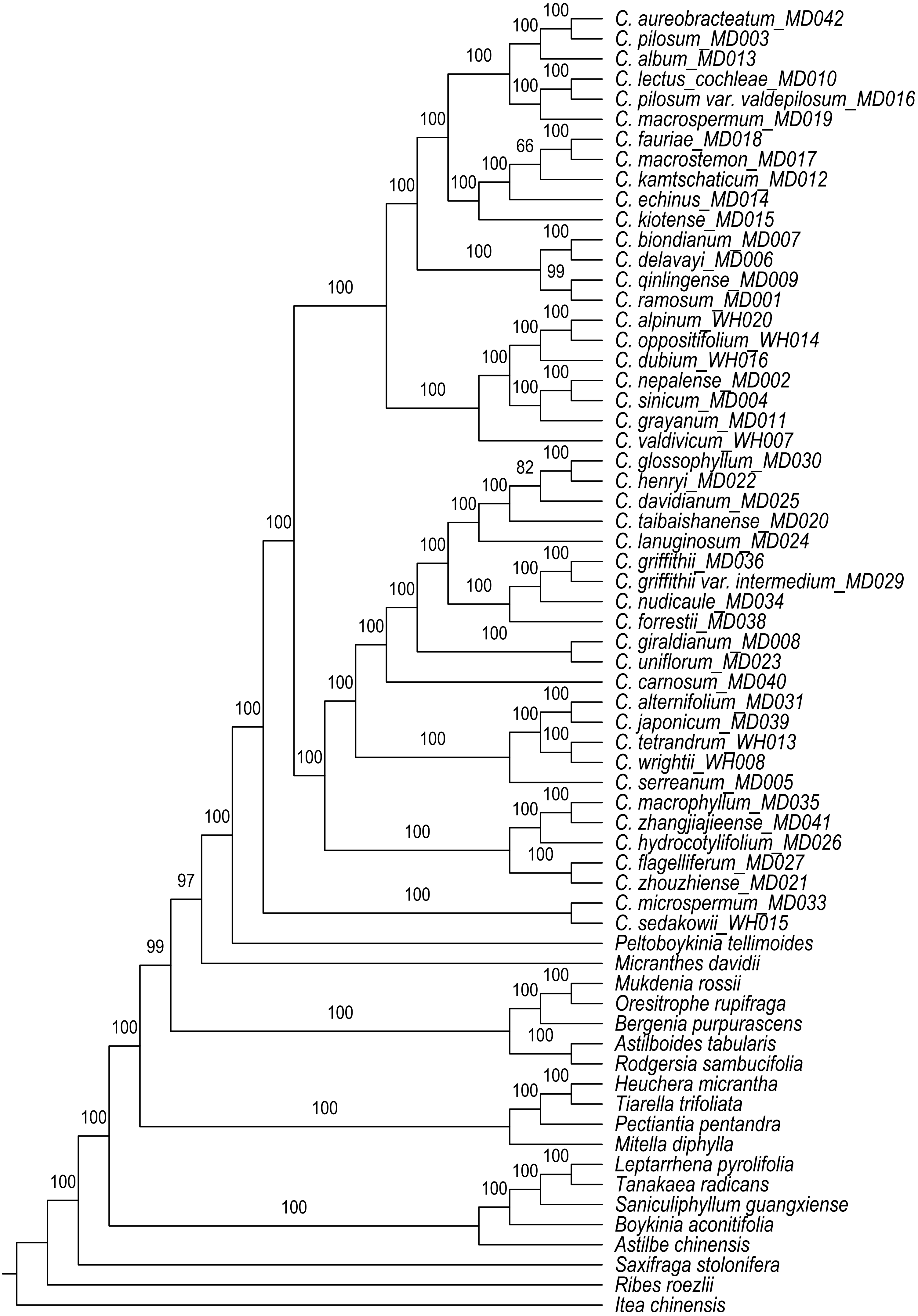

Figure S12. Phylogenetic tree of *Chrysosplenium* species using Maximum likelihood (ML) analyses based on cpPCGs+nrDNA matrix; The values on the nodes indicate the ML bootstrap support values.

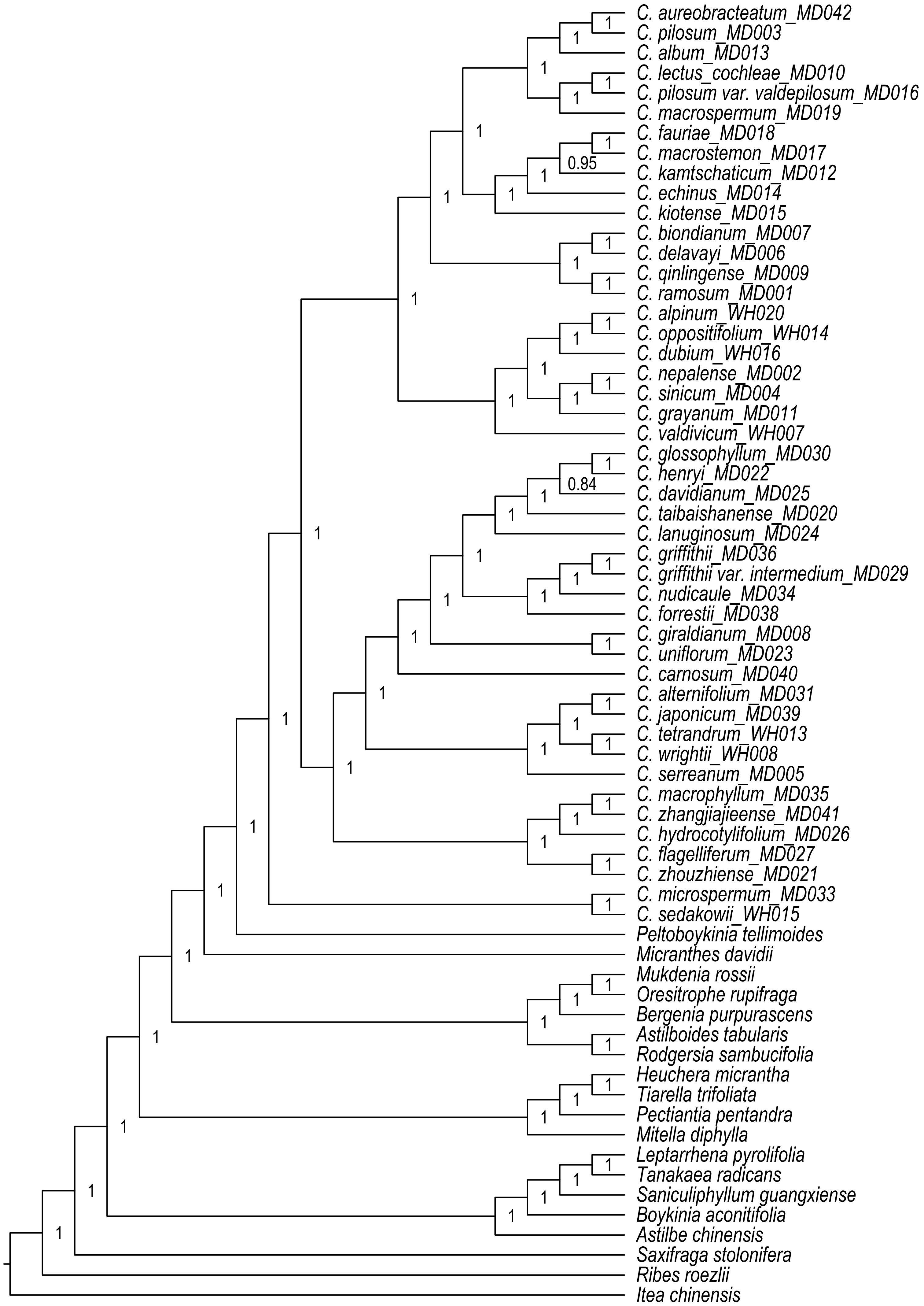

Figure S13. Phylogenetic tree of *Chrysosplenium* species using Bayesian inference (BI) analyses based on cpPCGs+nrDNA matrix; The values on the nodes indicate the BI posterior probabilities.

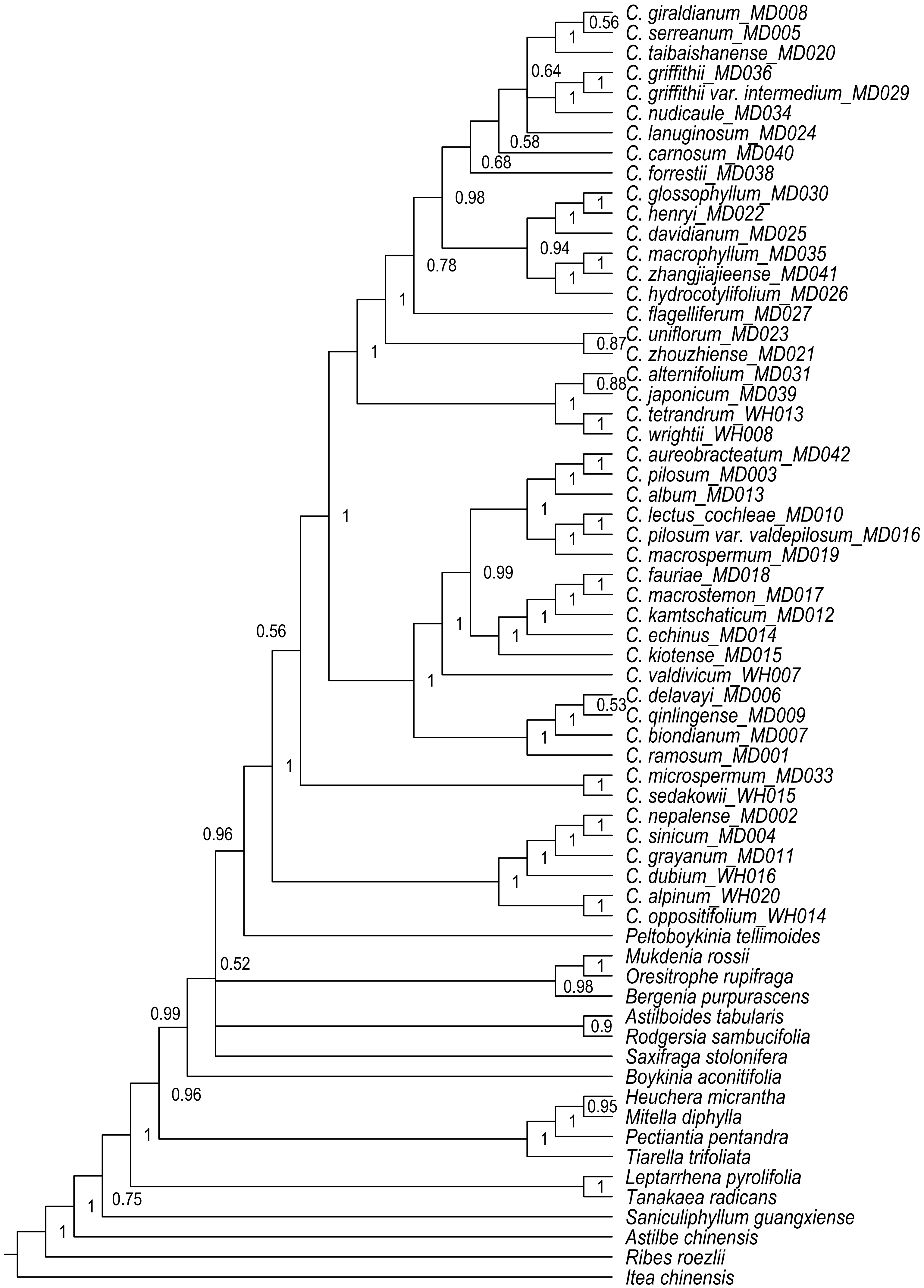

Figure S14. Phylogenetic tree of *Chrysosplenium* species using Bayesian inference (BI) analyses based on nrDNA matrix; The values on the nodes indicate the BI posterior probabilities.

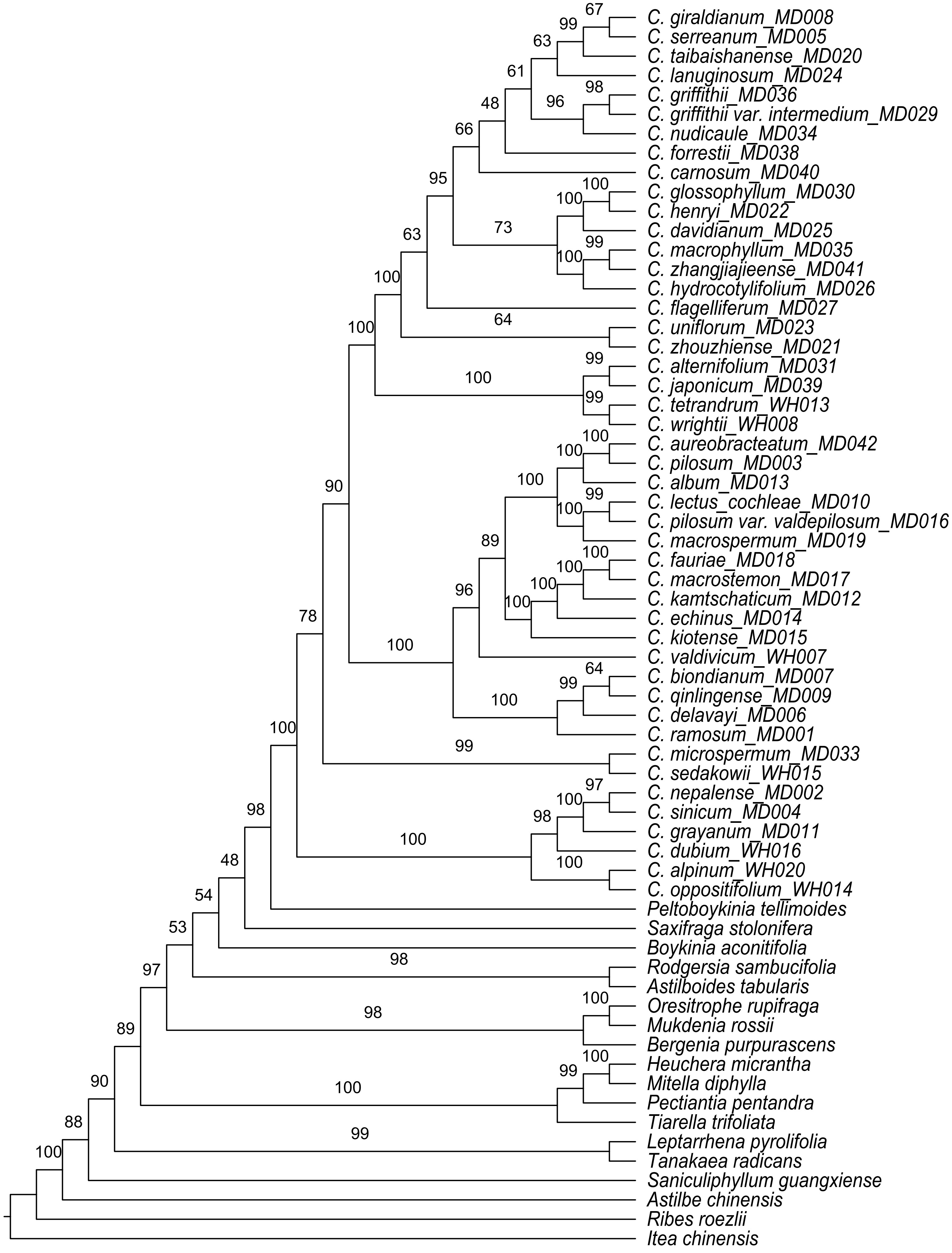

Figure S15. Phylogenetic tree of *Chrysosplenium* species using Maximum likelihood (ML) analyses based on nrDNA matrix; The values on the nodes indicate the ML bootstrap support values.
